# Molecular profiling of small renal masses: Current status and future directions

**DOI:** 10.4103/0970-1591.57922

**Published:** 2009

**Authors:** Balaji Kalyanaraman, Krishnanath Gaitonde, James F. Donovan

**Affiliations:** Division of Urology, University of Cincinnati College of Medicine, Cincinnati, Ohio, USA

**Keywords:** Molecular profiling, percutaneous renal biopsy, renal cell cancer, small renal masses

## Abstract

Small renal masses (SRMs) are renal tumors less than 4 cm in diameter. These account for the largest proportion of newly diagnosed renal cell cancers (RCC). Management of SRMs can be a dilemma if the patient is unfit to undergo partial nephrectomy. Molecular profiling enables better characterization of RCC and prediction of outcomes in terms of recurrence and progression. This article reviews the existing literature on molecular profiling of localized RCC, discusses limitations of molecular profiling, and presents the likely role that molecular profiling will play in guiding the treatment of SRMs.

## INTRODUCTION

An estimated 57,760 new cases of cancer of the kidney and renal pelvis will be diagnosed in the United States in 2009, an increase of 3000 cases compared to the previous year.[1,2] The overwhelming majority of these cancers will be renal cell cancers (RCCs). Widespread use of computed tomography (CT) for abdominal imaging has contributed to the increasing incidence of renal cell cancer by way of detecting incidental renal masses. For clinical staging purposes, localized renal cancers are classified by size as less than 4 cm (T1a), between 4 and 7 cm (T1b), and larger than 7 cm (T2). T1a tumors, also termed small renal masses (SRMs), account for the largest proportion of newly diagnosed renal cancers.[3] Surgical excision in the form of partial nephrectomy (PN) is the standard of care for T1a tumors and confers oncologic outcomes similar to radical nephrectomy (RN),[4] while offering distinct advantage in overall survival and noncancer-related mortality compared to RN.[5] However, patient factors such as cardiopulmonary comorbidities, tumor in a solitary kidney, or presence of chronic kidney disease may force the surgeon to consider alternative treatment options, namely active surveillance (AS) and thermal ablation (TA) (i.e., cryoablation and radiofrequency ablation). Although there have been no prospective studies to date comparing the efficacy of these treatment modalities to PN, meta-analysis by Kunkle *et al.* indicates that TA is associated with increased risk of local recurrence but no greater risk of metastases when compared with PN.[6]

In the process of determining the appropriate modality of therapy for a given SRM, it is also necessary to consider the natural history of that tumor. It is well known that RCCs are comprised of pathologically and genetically diverse population of cancers.[7,8] This implies that regardless of tumor size and histology, the molecular attributes of a RCC can determine its risk of recurrence and metastasis. Therefore, characterizing the molecular biology of a tumor can provide vital information to the surgeon to help stratify risk and thereby guide therapy.

## MOLECULAR PROFILING OF LOCALIZED RCC

Molecular profiling is defined as the classification of biological specimens, like tissues, blood, or urine, based on multiple molecule (like gene, protein, miRNA) expression patterns or genomic changes for diagnostic, prognostic, and predictive purposes.[9] Several molecular markers have been investigated for their role in the pathogenesis and progression of RCC. Those which have been consistently correlated with the prognosis of localized RCC will be discussed in this section with the emphasis on implications for recurrence and/or progression.

p53: Tp53 is a tumor suppressor gene that regulates cell-cycle at the G_1_-S transition point. The normal gene product, p53, induces apoptosis in proliferating cells that have undergone DNA damage. Mutations of p53 permit damaged cells to persist in the cell-cycle, thereby promoting carcinogenesis.[10] Mutant p53 protein has a long half-life, and therefore can be detected by immunohistochemistry as a nuclear stain.[11] In localized clear cell RCC (ccRCC), p53 mutation has been shown to negatively correlate with disease-free survival (DFS).[12] In the study incorporating various histologic forms of RCC, mutant p53 expression was found to be higher in non-ccRCC compared to ccRCC, with papillary RCC showing the highest expression.[12,13] In this study, p53-positive ccRCC had 56.3% progression after nephrectomy compared with 17.5% in p53-negative patients. In non-ccRCC, p53 expression did not have a statistically significant correlation with progression, although there was a positive trend. Of note, the median follow-up in this study was only 26 months and no cutoff was established for p53-positivity. Increasing the cutoff point for p53-positivity does not seem to alter the predicitive ability of the biomarker, as shown by Shvarts *et al*.[13] Conflicting evidence has been generated by Phuoc *et al*, whose data showed that mutant p53 expression in localized ccRNA was a significant prognostic factor on univariate analysis but not on multivariate analysis.[14]

### Ki-67:

Ki-67 is a nuclear antigen that is present in all cycling human cells and is a marker of cell proliferation.[15] Increased expression of Ki-67 has been associated with higher nuclear grade and worse prognosis in ccRCC.[16] Several recent studies suggest that Ki-67 expression could serve as an independent predictor of DFS in localized ccRCC on multivariate analysis[17,18] and that it may represent the true “molecular grade” of ccRCC.[18]

### IMP-3:

IMP-3 is a member of insulin-like growth factor (IGF) m-RNA binding protein family. It is expressed at low or undetectable levels in normal tissues, and overexpressed in cancers of pancreas, lung, colon, stomach, and soft tissue sarcomas.[19] In a study by Jiang *et al.*,[20] IMP-3 was overexpressed in 10% of stage-1 ccRCC. IMP-3 expression correlated negatively both with 5-year metastasis-free survival (IMP-3+ 44%; IMP-3- 98%) and overall survival (IMP-3+ 32%; IMP-3- 89%). External validation of this study confirmed the negative correlation of IMP3 expression with 10-year metastasis-free survival for TNM stage1 ccRCC.[21]

### Survivin:

Survivin is an antiapoptotic protein, which is overexpressed in almost all human cancers, including those of the kidney. Overexpression of Survivin has been shown to correlate negatively with DFS as well as overall survival in localized ccRCC.[22,23] Five- and ten-year progression-free survival in the Survivin-positive group was 58.8% and 45.9%, respectively, compared with 86.8% and 81.2% in the Survivin-negative group.[22] A smaller study showed that patients with Survivin-positive RCC had higher recurrence rate at 5 years compared with Survivin-negative RCC (72% vs. 93%), with no difference in overall survival.[23]

## MOLECULAR MARKERS AND PROGNOSTIC MODELS FOR LOCALIZED RCC

Several nomograms and algorithms exist to help guide the followup and treatment of RCC.[24–26] The current prognostic models use clinical variables such as tumor size, pathological stage, grade, nodal status, and histologic characteristics such as vascular invasion, to predict risk of recurrence and/or progression. The concordance index (CI), which represents accuracy of these models, ranges from 0.74 to 0.82. By integrating five molecular markers (Ki-67, p53, endothelial and epithelial VEGFR1, epithelial VEGF-D) with tumor T-stage and ECOG PS, Klatte *et al.* devised a nomogram to predict DFS in localized RCC, with CI of 0.90.[18] The accuracy of this nomogram was higher than that of T classification alone (CI 0.74) or UISS nomogram alone (0.78). Parker *et al.* have recently described a biomarker panel for ccRCC consisting of Ki-67, survivin, and B7-H1 (named Bioscore), which increased the CI of UISS from 0.774 to 0.819 and the CI of SSIGN algorithm from 0.821 to 0.837.[27] These demonstrate the contribution of molecular markers to enhance the power of clinical prognostic models in predicting long-term outcomes and risk of recurrent disease in localized ccRCC.

## LIMITATIONS OF MOLECULAR PROFILING OF RCC

Although biomarkers are a promising tool for the treatment and surveillance of RCC, it must be emphasized that the field of study is quite nascent and has numerous limitations. Most of the available data apply to ccRCC because it is the most commonly encountered variant. At best, limited information is available regarding the prognostic value of biomarkers in the context of papillary or chromophobe RCC – the data from ccRCC cannot be extrapolated to these other variants as they are biologically dissimilar.[7,8] The evaluation of biomarkers is predominantly semiquantitative and can be subject to observer bias. The determination of cutoff point for clinical significance is sometimes arbitrary and can alter the prognostic significance in the final analysis. In addition, the studies that do show prognostic significance can be considered for clinical application only after they are externally validated because almost all initial studies are single-institution based. Except for IMP-3, no other molecular marker has been externally validated to date.[21] Also, the molecular analyses carried out thus far have been on post-nephrectomy specimens and as such, biomarkers can be used only for surveillance purposes and not for primary treatment. There is an emerging need for research on preoperative molecular profiling of SRMs with the emphasis on percutaneous biopsy specimens. Finally, the cost of analyzing tumors for biomarkers in the clinical setting is prohibitive and these tests cannot be justified until their sensitivity and specificity significantly exceed those of current diagnostic methodologies.

## FUTURE DIRECTIONS

**Figure 1:**
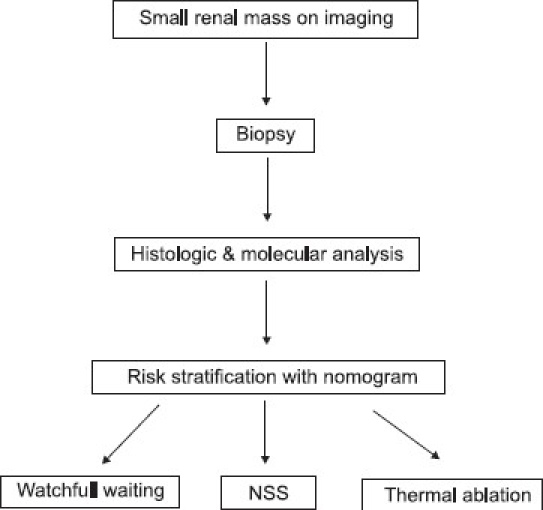
Future role of molecular profiling in the management of SRMs.

As mentioned earlier, one of the challenges in the treatment of RCC is to decide which subset of SRMs needs surgical intervention.[28] This is especially true as the population ages, and the surgeon is faced with comorbidities such as chronic kidney disease or solitary kidney in which case watchful waiting may be the best nephron-sparing strategy. Molecular profiling of percutaneous needle biopsy specimens could provide data on this population of RCC and potentially translate into clinically relevant nomograms. Recent studies on needle biopsy of SRMs for histopathologic diagnosis have reported sensitivities of 70–92% and specificity of 100% with no significant complications or needle-track seeding.[29,30] Therefore, percutaneous tumor biopsies can be considered safe and effective for SRMs.[31] High-throughput microarray analysis makes it possible to obtain molecular information from needle biopsy specimens[32] and such information could be used in nomograms to stratify risk accurately and to guide treatment. This likely evolution of management of SRMs is illustrated in Figure 1. In summary, molecular profiling of renal tumors is poised to take the leap from “bench to bedside.”[32]
